# Positive Interactions Between Lactic Acid Bacteria Could Be Mediated by Peptides Containing Branched-Chain Amino Acids

**DOI:** 10.3389/fmicb.2021.793136

**Published:** 2022-01-11

**Authors:** Fanny Canon, Valérie Briard-Bion, Julien Jardin, Anne Thierry, Valérie Gagnaire

**Affiliations:** UMR STLO, INRAE, Institut Agro, Rennes, France

**Keywords:** peptidomic, proteolysis, cross-feeding, *Lactococcus lactis* and *Enterococcus faecalis*, interactions

## Abstract

Lactic acid bacteria (LAB) are responsible for the sanitary, organoleptic, and health properties of most fermented products. Positive interactions between pairs of LAB strains, based on nitrogen dependencies, were previously demonstrated. In a chemically defined medium, using milk and lupin proteins as sole nitrogen source, two proteolytic strains were able to sustain the growth of non-proteolytic strains, but one did not. The objective of the present study was, thus, to determine which specific peptides were implicated in the positive interactions observed. Peptides produced and involved in the bacterial interactions were quantified using tandem mass spectrometry (LC-MS/MS). About 2,000 different oligopeptides ranging from 6 to more than 50 amino acids in length were identified during the time-course of the experiment. We performed a clustering approach to decipher the differences in peptide production during fermentation by the three proteolytic strains tested. We also performed sequence alignments on parental proteins and identified the cleavage site profiles of the three bacterial strains. Then, we characterized the peptides that were used by the non-proteolytic strains in monocultures. Hydrophobic and branched-chain amino acids within peptides were identified as essential in the interactions. Ultimately, better understanding how LAB can positively interact could be useful in multiple food-related fields, *e.g*., production of fermented food products with enhanced functional properties, or fermentation of new food matrices.

## Introduction

Lactic acid bacteria (LAB) nitrogen nutrition depends on a three-component proteolytic system, as LAB are auxotroph for numerous amino acids, of which the number and nature are species- and strain-dependent (Aller et al., 2014; Teusink and Molenaar, 2017). The proteolytic system, extensively described in *Lactococcus lactis*, is composed of a cell-envelop proteinase (CEP), transporters of oligo-, tri-, and dipeptides, *i.e.*, Opp, DtpT, and Dpp, respectively, and intracellular peptidases, and provides LAB all required amino acids (Savijoki et al., 2006). The proteolytic system of *L. lactis* has mostly been studied in milk, in which the growth of *L. lactis* has been shown to depend on the activity and type of the proteinase to exceed a bacterial count of 10^8^ colony-forming units (cfu)/mL (Juillard et al., 1995). Two types of proteinase have been described: P-_I_ type, which preferably hydrolyzes β-casein and, to a lesser extent, κ-casein, and P-_III_ type, which cleaves β-, κ-, and α_S1_-caseins equally well (Flambard et al., 1998). In addition, proteinase variants, due to point mutations affecting the CEP coding gene, also induced strain-dependent specificities of the casein hydrolysis (Ji et al., 2021). LAB strains can also show specificity when it comes to peptide transport, thus further intracellular peptide utilization. No generic rule has been strictly established but peptide length, hydrophobicity, or charge have been put forward for peptide transport into the cell (Juillard et al., 1998; Proust et al., 2019). The oligopeptide-binding protein OppA, for example, has a low affinity for short and negatively charged peptides (Detmers et al., 1998; Juillard et al., 1998; Doeven et al., 2004).

**FIGURE 1:**
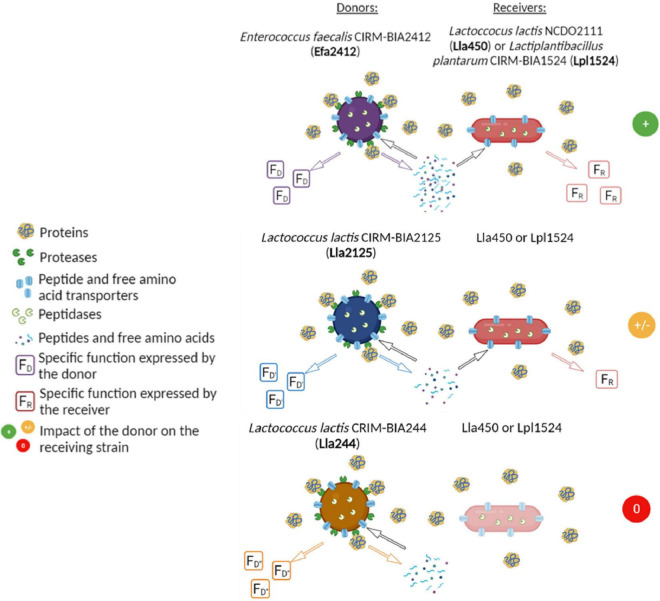
Interactions between proteolytic strains (=donors) and non-proteolytic strains (=receivers) observed in a chemically-defined medium containing caseins and lupin proteins as sole nitrogen sources (Canon et al., 2021).

Microbial positive interactions can involve cross-feeding and/or sharing of public goods, which often include nitrogen compounds (Canon et al., 2020). In LAB-yeast co-cultures, for example, amino acids provided by *Saccharomyces cerevisiae* have been shown to be responsible for the mutualistic or commensalistic interactions with *L. lactis* (Gobbetti, 1998; Ponomarova et al., 2017). In the co-culture of *Streptococcus thermophilus* and *Lactobacillus delbrueckii* subsp. *bulgaricus*, the two LAB species associated in yogurt, the sharing of peptides produced by *L. bulgaricus* was also shown to be essential for the interaction to occur, *i.e.*, for *S. thermophilus* growth. In fact, there was no interaction between *S. thermophilus* and *L. delbrueckii* when the former expressed a proteolytic activity (Settachaimongkon et al., 2014). The association of a proteolytic (prot^+^) *L. lactis* strain could provide the required nitrogen compounds to a CEP-deficient, non-proteolytic (prot^–^) *L. lactis* strain (isogenic or not) in co-culture thus resulting in positive interactions (Juillard et al., 1996). However, the association of prot^+^ and prot^–^ LAB strains does not systematically lead to the growth of the prot^–^ strain, even in the absence of inhibitors, as observed between *L. lactis* and *Leuconostoc mesenteroides* in milk (Bellengier et al., 1997). In another recent study, different interactions occurred in co-cultures of prot^+^ and prot^–^ LAB strains in a chemically-defined medium that contained milk and lupin proteins as sole nitrogen sources: the growth of prot^–^ strains were either not stimulated, stimulated, or strongly stimulated, while the growth of prot^+^ strain was not impacted (Figure 1; Canon et al., 2021). These interactions were mediated by peptides and/or amino acids, provided by the prot^+^ strains.

The aim of this study was thus to investigate why some prot^+^ strains were able to promote the growth of the prot^–^ strains while other prot^+^ strains did not, even when they exhibited a similar degree of proteolytic activity, *i.e.*, produced the same amount of peptides and amino acids. The same strains and co-cultures as presented in the study of Canon et al. (2021) were used. The strategy conducted was to characterize the peptides produced by the prot^+^ strains and identify the peptides preferably used by the prot^–^ strains in the co-cultures in which positive interactions were observed. To fulfill this aim, a peptidomic approach was conducted using tandem mass spectrometry. Our results suggest that branched-chain amino acids (BCAAs) are particularly involved in LAB interactions.

## Materials and Methods

### Bacterial Collection and Culture Conditions

Five mesophilic LAB strains were used, previously characterized for interactions in a study of Canon et al. (2021): *Lactiplantibacillus plantarum* CIRM-BIA1524 (Lpl1524), *Enterococcus faecalis* CIRM-BIA2412 (Efa2412), and *Lactococcus lactis* CIRM-BIA244 (Lla244), from the CIRM-BIA collection (International Center for Microbial Resources dedicated to bacteria of food interest, INRAE Rennes, France)^1^; *L. lactis* NCDO2125 (Lla2125) and *L. lactis* NCDO2111 (Lla450) from NCDO (National Collection of Dairy Organisms, now NC of Food Bacteria, Berkshire, United Kingdom). Efa2412, Lla2125, and Lla244 were considered as donor strains because of their proteolytic activity (prot^+^), whereas Lla450 and Lpl1524 were considered as receiver strains due to their lack of proteolytic activity (prot^–^) (Table 1).

**TABLE 1:** Origin and characteristics of the strains used.

*Genus*	*Species*	Strain number	Origin	Strain code	Proteolytic activity (Canon et al., 2021)
*Enterococcus*	*faecalis*	CIRM-BIA2412	NA	Efa2412	++^a^
*Lactococcus*	*lactis*	NCDO2125	Termite Gut	Lla2125	+^b^
*Lactococcus*	*lactis*	CIRM-BIA244	Raw milk	Lla244	+^c^
*Lactococcus*	*lactis*	NCDO2111	Pea	Lla450	−^d^
*Lactiplantibacillus*	*plantarum*	CIRM-BIA1524	Silage	Lpl1524	−^d^

*NA, non-available data.*

*^a^Favored strong positive interactions with non-proteolytic strains.*

*^b^Favored weak positive interactions with non-proteolytic strains.*

*^c^Did not favor positive interactions with non-proteolytic strains.*

*^d^Non-proteolytic strain.*

Strains were cultured in a chemically-defined medium (CDM) containing the main milk proteins, *i.e.*, caseins, and lupin proteins as sole nitrogen sources, for 22 h at 30°C, as described by Canon et al. (2021). Bacterial growth was monitored using compartmented chambers and lactococci and enterococci were enumerated on M17 and lactobacilli on MRS agar plates. To determine the type(s) of interaction occurring between donor and receiving strains, their growth in co-culture was compared to their growth in monoculture. We considered that the receiving strains were stimulated by the donor strains when an increase in bacterial growth rate and/or in maximal bacterial counts was observed in co-culture. Efa2412, Lla2125, and Lla244 favored strong, weak and null interactions with the receiving strains, respectively (Table 1). The three donor strains exhibited the same growth kinetics in monoculture and in co-cultures with a receiving strain (Canon et al., 2021). Samples were withdrawn from the compartmented chambers at the strategic time points of 6, 14, and 22 h. For the donor strains, 6, 12, and 22 h corresponded the middle exponential, early stationary, and late stationary growth phases, respectively. For the receiving strains, 6, 12, and 22 h corresponded to the start of growth, the middle exponential growth phase and the early stationary phase, respectively. The peptides produced were characterized using the monocultures of the donor strains, whereas the peptides used by the receiving strains were identified by comparing the co-cultures and the monoculture of the donor strains.

### Peptide Quantification by NanoLC-MS-MS

#### Sample Preparation

The CDM contained Tween 80, which interferes with the peptide ionization, rendering their analysis non-exploitable. A purification step was then needed and firstly done with detergent removal spin columns (87777, Thermo Scientific*™* Pierce, Waltham, MA, United States) according to the supplier’s protocol. Samples were further standardized prior to the injection to obtain a similar concentration of free NH_2_ groups, quantified with the o-phtaldialdehyde (OPA) method Church et al. (1983) adapted to microplate as described by Canon et al. (2021). Dilutions were performed in the separation buffer. For the samples that did not require dilution prior to injection, 0.2 μl of trifluoroacetic acid (TFA) at 5% (v/v) to reach a pH comprised between 2 and 3 as the buffer used for the peptide separation.

#### Separation and Ionization of Peptides

Mass spectrometry (MS) analyses were conducted as described by Deglaire et al. (2019). Briefly, a nano-RSLC Dionex U3000 system fitted to a Q-Exactive mass spectrometer (Thermo Scientific, San Jose, CA, United States) equipped with a nanoelectrospray ion source was used. Five μL of diluted samples were injected, concentrated on a μ-precolumn pepMap100 (C18 column, 300 μm i.d. × 5 mm length, 5 μm particle size, 100 Å pore size; Dionex, Amsterdam, Netherlands) and separated on a PepMap RSLC column (C18 column, 75 μm i.d. × 150 mm length, 3 μm particle size, 100 Å pore size; Dionex). Chromatography was performed at a flow rate of 300 nL/min using solvents A [2% (v/v) acetonitrile, 0.08% (v/v) formic acid and 0.01% (v/v) TFA in HPLC gradient grade water] and B [95% (v/v) acetonitrile, 0.08% (v/v) formic acid and 0.01% (v/v) TFA in HPLC gradient grade water]. The elution gradient first rose from 5 to 35% solvent B over 70 min, then up to 85% solvent B over 2 min before column re-equilibration. Separated peptides were ionized using the Proxéon source at an optimized tension of 2 kV. The mass spectra were recorded in positive mode using the m/z range 250–2,000 amu (atomic mass unit). The resolution of the mass analyzer for m/z of 200 amu was set in the acquisition method to 70,000 for MS and 17,500 for MS/MS. For each MS scan, the ten most intense ions were selected for MS/MS fragmentation. Ions with the same m/z value were then excluded from fragmentation for 20 s.

#### Identification of Peptides

Peptides were identified from the MS/MS spectra using the X!TandemPipeline software version 0.2.38 (Langella et al., 2017) against a ‘‘*Lupinus*’’ database (UNIPROT)^2^ composed of the 123 “reviewed” proteins and a homemade database containing major milk proteins. The following parameters were applied: non-specific enzyme cleavage; possible post-translational modifications: serine or threonine phosphorylation and methionine oxidation. Peptides identified with an *e*-value < 0.01 were automatically validated. The peptide false discovery rate was less than 0.1%.

#### Quantification of Peptides

**FIGURE 2:**
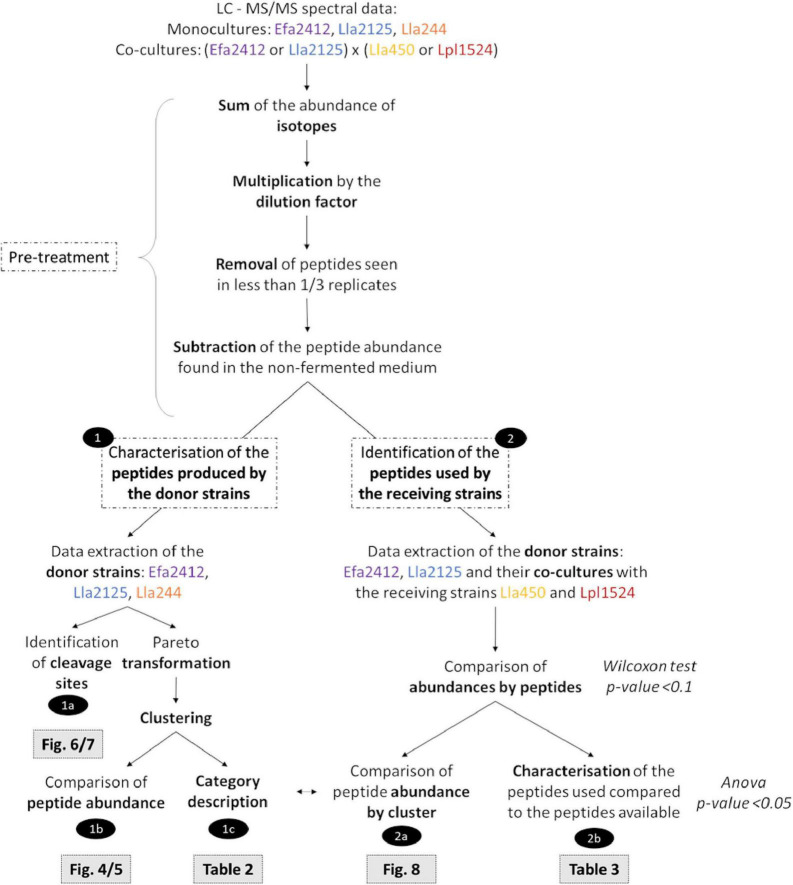
Workflow of the data treatment realized to compare the proteolytic profile of the three donor strains and to establish the characteristics of the peptides potentially involved in the interactions with the receiving strains.

Each identified peptide was quantified by label-free MS using the MassChroQ software (Valot et al., 2011) in all samples. A m/z width of 10 ppm was used to extract ion chromatograms (XIC) of peptides in time-aligned chromatograms and the area under the curve was then quantified. A pre-treatment of the dataset was performed (Figure 2). When a peptide was measured with several charge states (isotopes), all ion intensities were summed. The abundances were then multiplied by the dilution factor. The peptides found in at least one out of three replicates (3/9 for controls and 2/6 for samples) were considered. Abundances of the peptides found in the non-cultured CDM were subtracted from the abundances of the cultured samples (Figure 2).

#### Biochemical Characteristics and Composition of the Peptides

The isoelectric point, the molecular weight and hydrophobicity were determined for each peptide. The peptide composition (expressed both in number and percentages of amino acid residues) was also described using different categories: Essential amino acids for LAB [gathering the amino acids: arginine (R), isoleucine (I), leucine (L), valine (V), asparagine (N), tryptophan (W), tyrosine (Y), threonine (T), phenylalanine (F), serine (S), methionine (M), histidine (H), and glutamic acid (E)]; Non-polar [glycine (G), alanine (A), V, L, R, and I]; Polar non-charged [S, T, cysteine (C), proline (P), N, and glutamine (Q)]; Aromatic (F, W, and Y); Small [A, aspartic acid (D), N, C, G, P, S, T, and V)]; Tiny (A, C, G, S, and T); Aliphatic (A, I, L, and V); Charged [D, E, H, lysine (K), and R]; Acidic (D and E); Basic (H, K, and R); Branched-chain (I, L, and V); Hydrophobic (I, L, V, F, W, and C); Sulfurous (M and C).

#### Peptide Alignments on Parental Proteins

Peptide alignments were performed with the online software Peptigram^3^ [Manguy et al., 2017; Figure 2(1a)]. The sequences of the referenced proteins contain the signal peptide, thus implicating a shift in the lecture frame.

### Statistical Analysis

The analyses were all performed using R software, version 4.0.5 (R Core Team, 2020). The global strategy is depicted in Figure 2.

#### Heatmap and Clustering of the Peptides Produced by the prot^+^ Strains

Data from the monocultures of the donor strains were extracted from the whole dataset. Data of peptide abundances were log-transformed and Pareto-scaled using the function scaling of the package MetabolAnalyze. According to van den Berg et al. (2006), Pareto scaling consists in dividing the log of the abundance of one peptide by the square root of the standard deviation of this same peptide in all samples:


$$x_{p}=\frac{l\,o\,g\,\left(x_{i}\right)}{\sqrt{s}}$$

This scaling method permits to reduce the impact of high abundance values while keeping a good sensibility regarding important variations (van den Berg et al., 2006). The heatmap of the abundance of peptides for each donor strain and time was performed using the function heatmap 0.2 of the R package gplots (Figure 2). The clustering of the peptides was performed using the function hclust of the R package stats based on the minimum within-cluster variance Ward’s agglomeration.

#### Comparison of Peptide Abundances by Cluster

Non-parametric tests were performed to compare the abundance of peptides between donor strains and at the different times because of the high percentage (around 82% after filtering) of null values in the dataset, which is a common feature of peptidomic analyses. To compare the abundance of peptides during incubation time for one given strain, a Friedman test was performed on repeated measures (Abundance ∼ Time | Peptides) using the function friedman.test of the R package stats. When significant differences were found, a Conover *post hoc* test with a Holm adjustment of the *p*-values was performed using the function *post hoc*.friedman.conover.test of the R package PMCMR (*p*-value < 0.05). To compare the abundance of peptides produced by the donor strains at one given time a Kruskal-Wallis test (Abundance ∼ Strain) was performed using the function kruskal.test of the R package stats. When significant differences were found, a Wilcoxon *post hoc* test with a Benjamini-Hochberg adjustment of the *p*-values was performed using the function pairwise.wilcox.test of the package stats [*p*-value < 0.05; Figure 2(1b)].

#### Category Description of the Clusters

Cluster description was performed with the catdes function of the R FactoMiner package using the biochemical characteristics and composition of the peptides. A Chi-square test performed for qualitative variables (protein origin and cleavage sites), and a one-way analysis of variance for quantitative variables (isoelectric point, molecular weight, percentages in certain amino acids). V-test indicated whether the modality category was significantly overrepresented (v > 2) or underrepresented (v < −2) [Figure 2(1c)].

#### Comparison of Abundance by Peptide

To establish which peptides were used by the receiving strains, we compared the abundance of each peptide between the monoculture of a donor and a co-culture of this donor strain with a receiving strain. A Wilcoxon test was performed with the wilcox.test function of the R package stats. The *p*-value was set at 0.1 [Figure 2 (part 2)].

#### Comparison Between the Peptides Used by the prot^–^ Strains and the Overall Peptides Produced by the prot^+^ Strains

The peptides which showed a significant decrease at the previous step were characterized by using the same characteristics as for the category description. The cluster number of the peptides used was retrieved [Figure 2(2a)]. The peptides used by the receiving strain were compared to the overall peptides produced by the donor strain for each criterion using an analysis of variance, performed with the function aov of the R package car [*p*-value < 0.05; Figure 2(2b)].

## Results

### Clustering of Peptides Produced by the prot^+^ Strains at Each Time Point

**FIGURE 3:**
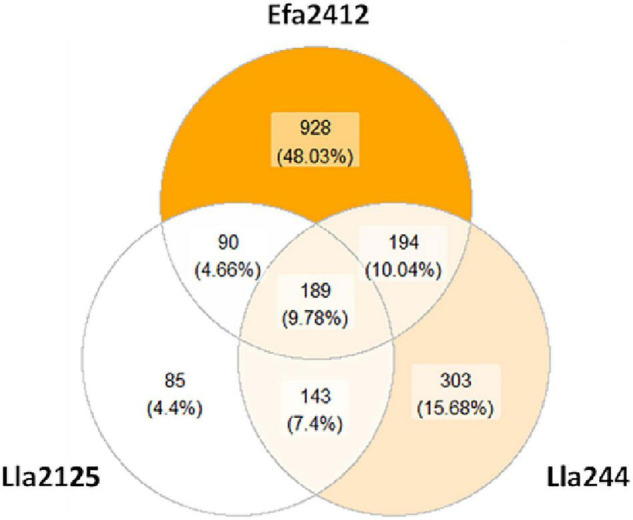
Venn diagram of the peptides produced by the three donor strains throughout the 22 h of culture. The color scale is correlated with the number of peptides counted.

A total of 1,932 unique peptides were identified as produced by the three donor strains, 65% of which were of bovine milk origin. Their size ranged from 6 (minimum size considered) to 50 amino acid residues. Efa2412 markedly differed from the two other donor strains by both a higher abundance and a higher number (1401) of peptides. The two *L. lactis* strains exhibited similar profiles, with 829 and 507 peptides produced by Lla244 and Lla2125, respectively (Figure 3). Almost half of the peptides were only produced by Efa2412. 10% of the peptides were produced by the three donor strains. Some peptides were common between Efa2412 and each of the *L. lactis* strains.

**FIGURE 4:**
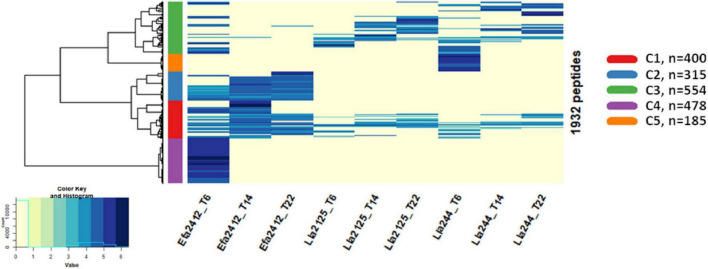
Heatmap of the abundance of the peptides produced by the three donor strains after 6, 14, and 22 h of culture. The color scale represents the mean abundance (after the log Pareto transformation) of the identified peptides.

The peptides produced were distributed in five clusters according to their abundance at the three time points for the three donor strains (Figure 4). Two clusters (C2 and C4) gathered Efa2412-specific peptides and one cluster (C5) Lla244-specific peptides, whereas no cluster of Lla2125-specific peptides was identified. Two clusters (C1 and C3) gathered peptides produced by all three donor strains (Figure 4). Concerning these two clusters of non-specific peptides, Efa2412 produced significantly more peptides in the cluster 1 compared to both *L. lactis* strains, whereas Lla244, followed by Lla2125, produced significantly more peptides in cluster 3.

**FIGURE 5:**
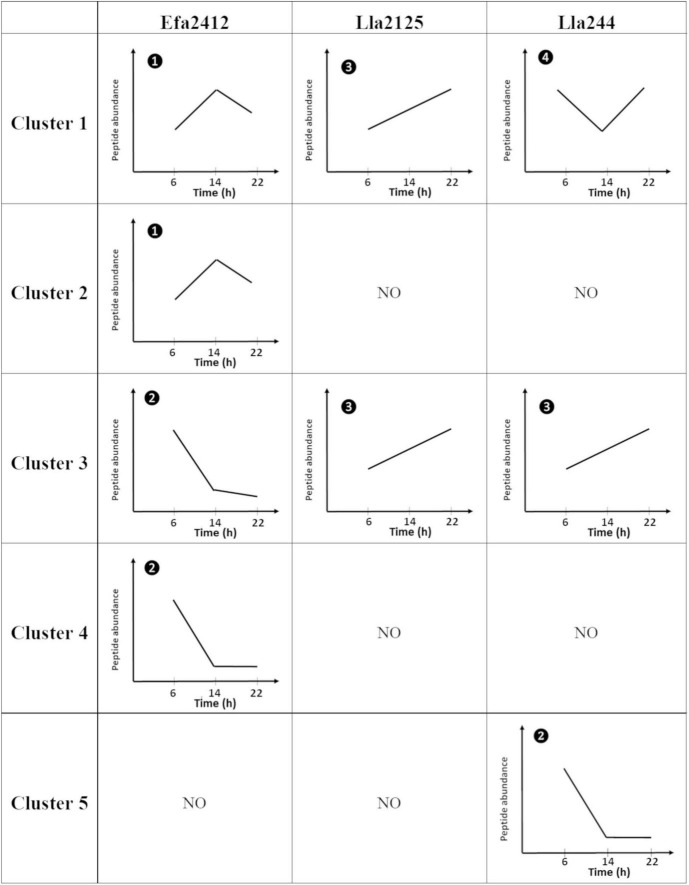
Representation of the global kinetic profiles of the peptides according to the five clusters depicted in Figure 4 and the three donor strains. The variations represented were statistically significant for each time (Friedman test, *p*-value < 0.05). ➊ Peptides accumulated then decreased; ➋ peptides decreased until they are no more detected; ➌ peptide continuously accumulated; ➍ peptides decreased then accumulated. An accumulation means that peptides were produced faster than they were further cleaved or/and used, while a decrease means that they were cleaved or/and used faster than they were produced. NO, not observed.

Different kinetics of peptide abundance were observed depending on the clusters and the donor strains. Overall, the abundance of the peptides produced by Efa2412 increased from 6 to 14 h then decreased up to 22 h (C1 and C2) or decreased between 6 and 22 h (C3 and C4). For Lla2125 the abundance of the peptides kept increasing between 6 and 22 h (C1 and C3) (Figure 5). The same profile was observed for the peptides produced by Lla244 belonging to C3, whereas the peptides found in C1 increased then decreased, and the peptides from C5 were only found at 6 h.

### Category Description of the Clusters According to the Physicochemical Properties and Composition of the Peptides

The results of the category description show that the five clusters contained peptides that exhibited contrasted characteristics (Table 2). C1 was similar to C2 and both contained peptides with opposite characteristics compared to C4. C1 and C2 mostly contained small peptides, negatively charged according to the isoelectric point and the high proportions in acidic residues, while, in contrast, the cluster C4 mostly contained large and positively charged according to the isoelectric point and the high proportions in basic residues. Cluster C4 also contained peptides with higher proportions in hydrophobic, aliphatic, aromatic, and sulfurous residues. Moreover, clusters C3 and C5 also exhibited contrasted profiles: C3 was mostly composed of peptides with higher proportions in hydrophobic, and aliphatic residues and low proportions in essential and polar residues, contrary to C5. Regarding their origin, peptides derived from lupin proteins were in significantly high proportions in C5 and in C1 and less in C3 and C4, as opposed to the peptides derived from caseins.

**TABLE 2:** Description of the clusters according to the peptide characteristics and the amino acid (AA) composition.

	C1	C2	C3	C4	C5	Counted amino acids
**Quantitative characteristics**						
mw						
pI						
Hydrophobicity						
Hydrophobic AA (%)						G + A + I + L + V + P + F + W + M
Aliphatic AA (%)						G + A + I + L + V + P + M
Non-polar AA (%)						G + A + I + L + V + M
Branched-chained AA (BCAAs, %)						I + L + V
Essential AA (x13, %)						R + I + L + V + F + Y + W + M + S + T + N + H + E
Essential AA (x7, %)						R + I + L + V + M + H + E
Sulfurous side chain (%)						C + M
Polar non-charged AA (%)						S + T + N + Q + C + Y
Aromatic AA (%)						F + Y + W
Tiny side chain (%)						G + A + C + S + T
Small side chain (%)						G + A + V + D + P + S + T + N + C
Charged AA (%)						D + E + R + K + H
Acidic AA (%)						D + E
Basic AA (%)						R + K + H
**Qualitative characteristics: protein-derived peptides**						
Lupin proteins						
β-conglutin						
α-conglutin						
γ-conglutin						
Caseins						
β-casein						
α_S1_-casein						
α_S2_-casein						
κ-casein						

*Colored cells indicate significant over- (in blue) or under-represented (in red) criteria between clusters. Dark blue = v test > 5, light blue = 2 < v test > 5, dark red = v test < -5, light red = -2 < v test > -5.*

### Origin of Peptides: Proteins Cleaved by prot^+^ Strains and Preferred Cleavage Sites

**FIGURE 6:**
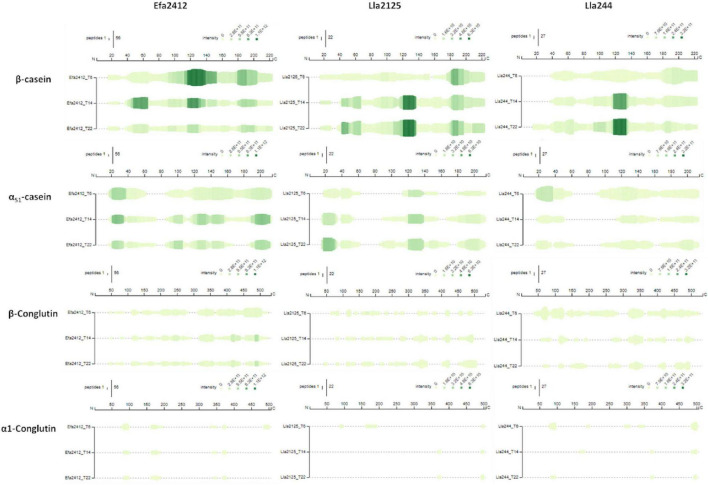
Peptide profiles on the β-casein (CASB), α_S1_-casein (CASA1), α_1_-conglutin (CONA1), and β-conglutin (CONB2). The color scale represents the summed abundances and is normalized for the four proteins for each donor strain (Note that it differs for a given protein, between the three strains). The size of the vertical bars represents the number of peptides considered for each amino acid in the sequence. The peptides were aligned on the protein sequences provided by the database Uniprot (uniprot.org) using the online software Peptigram (http://bioware.ucd.ie/peptigram/). The identifiers are: F5B8V6 for CONA1, Q6EBC1 for CONB2, P02662 for CASA1, and P02666 for CASB. The signal sequence is included.

The capacity of the donor strains to hydrolyze the major proteins of the medium was investigated as shown in Figure 6. The three donor strains hydrolyzed β-casein entirely and most of the α_s1_-casein. Compared to milk proteins, they hydrolyzed the lupin proteins far less, especially α_1_-conglutin. Most of the sequence of α_1_-conglutin was not attacked by the proteinases of any donor strain. Overall, Efa2412 hydrolyzed all proteins more than Lla2125 and Lla244 as the summed abundances were 10 times higher for each protein. Proteins were also hydrolyzed faster by Efa2412 as the summed abundances were higher after 6 h of culture. For all proteins, the summed abundance decreased between 14 and 22 h in Efa2412 culture, while it increased in Lla2125 and Lla244 cultures. The proteolytic profiles of Lla2125 and Lla244 were very similar, although Lla244 showed higher abundances of peptides and a higher hydrolysis of β-conglutin.

**FIGURE 7:**
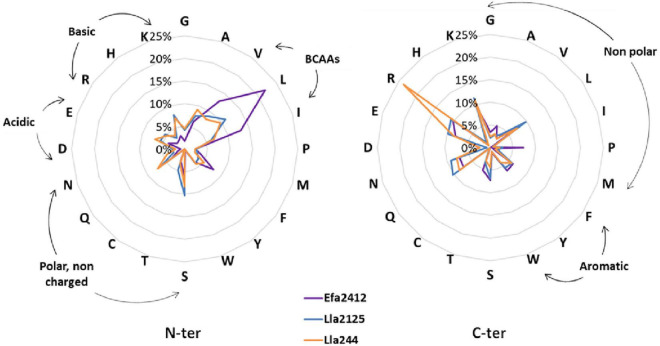
Profile of the cleavage sites observed for the three donor strains: amino acid frequencies at N-ter and C-ter positions of all the peptides produced by the strains Efa2412, Lla2125, and Lla244, independently on the parental proteins and the time. *n* = 1401 for Efa2412, *n* = 507 for Lla2125, and *n* = 829 for Lla244. BCAAs = branched-chain amino acids.

The cleavage sites of the peptides produced by Efa2412 mostly involved the three branched-chain amino acids (BCAAs), I, L, and V, with 13, 22, and 13% of N-ter amino acids, respectively, against 6, 11, and 9% for Lla2125 and 7, 10, and 8% for Lla244. The cleavage site profiles of N-ter amino acids of Lla2125 and Lla244 were highly similar (Figure 7). The cleavage sites profiles were similar for the three strains for the C-ter position, except for Lla244, which produced twice more peptides with arginine (R), compared to Efa2412 and Lla244.

### Peptides Used by the prot^–^ Strains

The preferred peptides used by two receiving strains, Lla450 and Lpla1524, were investigated by comparing the co-cultures in which they grew, *i.e.*, the ones with the donor strains Efa2412 and Lla2125, with the monoculture of the donor strains. The abundance of each peptide produced by the donor strains was compared to its abundance in the co-culture with a receiving strain (Table 3). Peptides of which the abundance significantly decreased were considered as used by the receiving strain. They represented only 3–15% of all peptides produced. Among the peptides produced by Efa2412, Lla450 preferably used large peptides containing more non-polar, small side chain, hydrophobic, branched-chain, aliphatic residues, as well as amino acids considered as essential for the growth of *L. lactis* (Cocaign-Bousquet et al., 1995; Aller et al., 2014; Table 3). Similar tendencies were found with Lpl1524 but no significantly different characteristics were observed. Among the peptides produced by Lla2125, Lla450 preferably used peptides containing non-polar and branched-chain amino acids, as well as amino acids considered as essential (Table 3). Lpl1524 preferably used peptides containing less acidic and more hydrophobic amino acids (Table 3).

**TABLE 3:** Comparison of peptide abundances in the monocultures of the donor strains Efa2412 and Lla2125 and their co-cultures with the receiver strains Lla450 or Lpl1524.

Cultures	Time	Number of peptides produced by the donor strain	Number of peptides decreasing significantly compared to the peptides in the donor monoculture (*p*-value < 0.1)	Differences in properties of the peptides used compared to the pool of peptides available (*p*-value < 0.05)	Mean values of peptides properties	
					For the peptides produced	For the peptides used
Efa2412 × Lla450	T6	1113	32 (3%)	NS	NS	NS
	T14	657	62 (9%)	Length	15.8	18.3
				mw	1,826.7	2,091.4
				BCAA	3.0	3.6
				Essential (x13)	9.8	11.4
				Essential (x7)	6.0	6.8
				Hydrophobic	6.9	8.2
				Non-polar	4.6	5.6
				Polar non-charged	4.5	5.5
				Tiny	3.1	3.8
				Aliphatic	6.3	7.6
				Small	7.3	8.7
	T22	531	78 (15%)	Length	15.8	18.6
				mw	1827.3	2163.2
				BCAA	3.1	3.9
				Essential (x13)	9.8	11.5
				Essential (x7)	6.1	7.9
				Acidic	2.3	3.1
				Non-polar	4.7	5.8
				Polar non-charged	24.3	28.5
Efa2412 × Lpl1524	T6	1,113	Lpl1524 counts < 5.10^7^ cfu/mL			
	T14	657	61 (9%)	NS	NS	NS
	T22	531	49 (9%)	NS	NS	NS
Lla2125 × Lla450	T6	199	Lla450 counts < 5.10^7^ cfu/mL			
	T14	265	21 (8%)	Essential (x7)	6.8	8.5
				Non-polar	5.3	7.3
				BCAA	3.7	5.2
	T22	322	26 (8%)	Essential (x7)	6.7	8.4
				Non-polar	5.1	6.8
Lla2125 × Lpl1524	T6	199	Lpl1524 counts < 5.10^7^ cfu/mL			
	T14	265	20 (8%)	Hydrophobic	8.4	11.0
				Acidic	13.2	8.1
	T22	322	10 (3%)	NS	NS	NS

*The last two columns represent the characteristics of the peptides significantly less abundant in the co-culture compared to the pool of peptides provided by the donor. NS means that the characteristics of the peptides used did no significantly differ from that of the total peptide available.*

**FIGURE 8:**
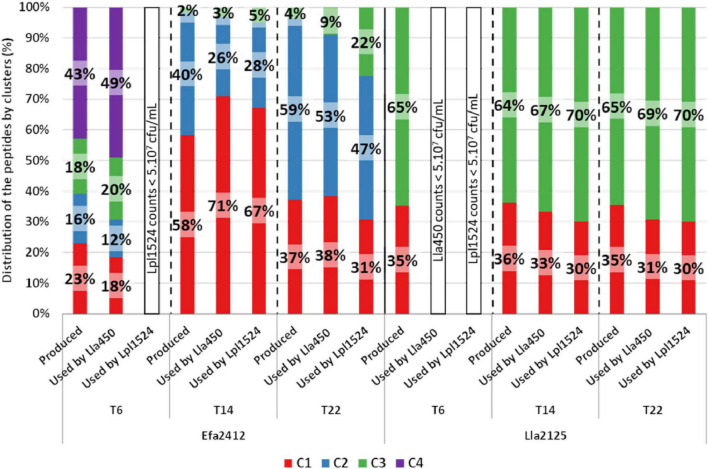
Distribution of the peptides by clusters either (i) produced by the prot^+^ strains Efa2412 and Lla2125 or (ii) used by the prot^–^ strains Lla450 and Lpl1524 in co-culture with Efa2412 or Lla2125. Peptides considered as used by the prot^–^ strains were the ones for which the abundance was significantly lower in co-culture than in monoculture of the prot^+^ strain (Wilcoxon test, *p*-value < 0.1).

The peptides that were less abundant in Efa2412 co-cultures compared to Efa2412 monoculture were mostly found in clusters C4 at T6, C1 at T14, and C2 at T22, for both prot^–^ strains (Figure 8). Peptides significantly less abundant in Lla2125 co-cultures compared to Lla2125 monoculture were found in cluster C3 (Figure 8). Overall, clusters C4 and C3 were more represented in the peptides used compared to the peptides produced, in contrast to cluster C2 (Figure 8).

## Discussion

We previously investigated interactions based on cross-feeding of nitrogen compounds in co-cultures that associated proteolytic (prot^+^) and non-proteolytic (prot^–^) LAB strains (Canon et al., 2021). These interactions only concerned prot^–^ strains, which grew differently according to the prot^+^ strains they were associated with, while prot^+^ strains grew similarly in mono- and co-culture. We showed that the most stimulatory prot^+^ strain was a highly proteolytic *E. faecalis* strain (Efa2412), which produced high concentrations in amino acids and peptides (33.4 and 48.8 mg NH_2_/L, respectively) (Canon et al., 2021). In contrast, a moderate or even a null stimulation of prot^–^ strains was exerted by the two *L. lactis* strains Lla2125 and Lla244, respectively, although they produced peptides (4.0 and 5.8 mg NH_2_/L, respectively) and high concentrations in amino acids (37.8 and 21.9 mg NH_2_/L, respectively) (Figure 1; Canon et al., 2021).

In the present study we aimed to characterize in depth the peptide profile produced by the prot^+^ strains in order to have clues for understanding how prot^+^ strains can stimulate or not the growth of prot^–^ strains, using a peptidomic approach (Figure 2). First, the peptide profiles of the prot^+^ strains allowed us to discriminate the three prot^+^ strains, by: (i) clustering peptides according to their abundance at each time point for each prot^+^ strain, and (ii) describing the specificities of each cluster in terms of amino acid composition, peptide length, and isoelectric point. Peptides were also aligned on the parental proteins to identify cleavage sites as well as the variable capacity of the prot^+^ strains to hydrolyze the major proteins of milk, *i.e.*, caseins and of lupin. Second, we identified the peptides preferably used by the prot^–^ strains in the co-cultures for which positive interactions had been previously observed.

### Efa2412, the Most Stimulating prot^+^ Strain, Has a Proteolytic Profile Very Distinct From That of Lla2125 and Lla244

Efa2412 was previously shown as the prot^+^ strain that induced the highest positive interaction with prot^–^ strains (Figure 1; Canon et al., 2021). In this study, we showed that this strain produced more peptides in terms of number and abundance, compared to both *L. lactis* prot^+^ strains (Figures 3–6), in agreement with the results of NH_2_ group quantification previously observed in the same cultures (Canon et al., 2021). In general, the abundance of peptides kept decreasing for Efa2412 whereas it kept increasing for Lla2125. Lla244 showed a hybrid profile: part of the peptides produced decreased in abundance during time while the others increased (Figures 4–6). These differences in peptide production could be explained by the fact that *E. faecalis* has two supplementary proteinases: gelatinase (GelE) and glutamyl endopeptidase (serine proteinase V8, SprE). Both these proteinases are excreted in the medium (Worsztynowicz et al., 2019) and could cleave proteins and peptides more extensively than the CEP of the *L. lactis* strains do. Another hypothesis to explain the greater proteolytic activity of *E. faecalis*, compared to that of *L. lactis*, is that the proteolytic system of *L. lactis* is repressed by the intracellular pool of branched-chain amino acids, regulated by the repressor CodY (Guédon et al., 2001). This study shows, for the first time, the cleavage sites of *E. faecalis* proteinases on milk and lupin proteins. Thus, we showed that Efa2412 preferably cleaved proteins before the branched-chain amino acid residues, as indicated by their high frequency in the N-ter position of the peptides produced (Figure 7). The two *L. lactis* prot^+^ strains hydrolyzed β- and α_s1_-caseins, suggesting that their CEP was a P_III_ proteinase type (Figure 6; Ji et al., 2021). The peptides early and specifically produced by Efa2412 were characterized by a high molecular weight, high amounts in hydrophobic, non-polar, branched-chain and aliphatic residues (cluster C4, Table 2). In contrast, the specific peptides produced by Lla244 (cluster C5), which did not stimulate the growth of prot^–^ strains, had the opposite characteristics (Table 2).

### The Study of Peptide Intake in a Co-culture Set-Up: An Unprecedented Approach

The novelty of our approach relies on the fact that we studied the peptide intake of LAB strains in a dynamic co-culture set-up. Up to now, peptide intake of one strain of *S. thermophilus* cultured in a yeast extract has been previously investigated using LC-MS-MS and it was shown that *S. thermophilus* used preferably short peptides with a global positive net charge and a higher proportion of hydrophobic residues (Proust et al., 2019). In another study, in co-culture with *Levilactobacillus brevis, S. thermophilus* was able to break down caseins to provide free amino acids and peptides to sustain the growth of *L. brevis* (Xiao et al., 2020). However, the type of peptides preferably used by *L. brevis* was not investigated yet.

In our study, we attached importance to identify the type of peptides used by the prot^–^ strains, and this is why we compared the abundance of each peptide between, on the one hand, the monoculture of each prot^+^ strain and, on the other hand, the co-culture of this strain with each prot^–^ strain. Such an approach had not yet been applied, to our knowledge, in bacterial co-culture systems, but was previously used to study peptide release kinetics during digestion (Deglaire et al., 2019). Our approach was a challenge since mass spectrometry approaches are generally prone to a lot of variability as discussed in recent studies (Guillot et al., 2016; Bingeman et al., 2017). Moreover, as we chose to grow bacteria in co-cultures and not in sequential cultures, which would have been simpler, we further increased this level of variability. We made this choice to better mimic the reality of fermented foods, as the pool of peptides produced by prot^+^ LAB strains differed, in nature and abundance, during the time course of culture (Figures 4–6). Actually, in sequential cultures, prot^–^ strains would only have benefited from the peptides available at the end of the prot^+^ strain growth. In our case, we assumed that the peptides used by prot^–^ strains were predominantly in the pool of peptides for which the abundance was significantly inferior in the co-culture prot^+^/prot^–^ compared to the monoculture of prot^+^.

### The prot^–^ Strains Preferred Peptides Containing High Amounts in Branched-Chain Amino Acid Residues

In our study, the peptides used by the prot^–^ strains only refer to the peptides transported by the Opp system, *i.e.*, with a peptide length over six residues, corresponding to the minimal length required for peptide identification. Thus, the di- and tripeptides, which are transported by DtpT or Dpp, could not be studied although these latter play an important role in nitrogen nutrition (Foucaud et al., 2001; Saguir et al., 2008) and could have been part of the overall peptides produced. Two complementary approaches were used to shed light on the types of peptides used by the prot^–^ strains. The first one was to identify the cluster associated with the peptides considered transported by the prot^–^ strain (Figure 8). The second one was to compare the characteristics of the peptides used at each time to all peptides produced by the only two prot^+^ strains that stimulated the growth of the prot^–^ strains (Table 3).

The peptides used by the prot^–^ strains were not restricted to specific clusters, but preferences for peptides from clusters C4 and C3 were observed (Figure 8). These two clusters were characterized by a high percentage in hydrophobic and aliphatic residues. The prot^–^ strain Lla450 preferred large peptides (∼2,000 kDa) containing more amino acids considered as essential for LAB growth, as well as those containing more hydrophobic, aliphatic, non-polar, and branched-chain residues (Table 3).

Four characteristics of the peptides used by the prot^–^ in co-culture with Lla2125 were identified as possible stimulator factors, and were similar with the ones identified with Efa2412. These concern peptides containing high amounts in (i) branched-chain, (ii) non-polar, and (iii) hydrophobic and (iv) amino acids considered as essential for LAB. The common point of these four characteristics was the presence of the hydrophobic branched-chain amino acids I, L, and V. Our results agree well with several previous studies that describe peptide transport in *L. lactis* (Doeven et al., 2005; Berntsson et al., 2011), in which hydrophobic residues were shown to bind preferentially to the oligopeptide-binding protein and thus to be preferentially transported via the Opp system. In our study, the peptide length appeared as a selectivity factor for peptide transport for *L. lactis* but not for *L. plantarum*, which agrees with the fact that the Opp system of *L. lactis* can take in peptides containing up to 35 amino acid residues. The importance of the peptide charge for peptide transport by *L. lactis* was questioned in a recent article of Proust et al. (2019). In our study, the peptide charge did not appear as a prevailing selectivity criterion for peptide transport.

The peptides used by Lpl1524 were less characterized as they only slightly differed from the global pool of peptides produced by the prot^+^ strains. With Lla2125, Lpl1524 only showed preferences for peptides containing more hydrophobic and less acidic residues. *L. plantarum* exhibit more auxotrophies in amino acids than *L. lactis* (Aller et al., 2014; Ma et al., 2016). We thus hypothesize that its peptide transporters are less selective and/or that *L. plantarum* preferably internalize di- or tripeptides.

Our results suggest a causal connection between strong positive interactions and the production/use of branched-chain amino acid-containing peptides. To support further this assertion, peptides of different length and sequence, containing branched-chain amino acids or not, could be added in the CDM to directly investigate their impact on the growth of receiving strains. It would also help deciphering the importance of di- and tripeptides in the interactions.

### The Shortage of Peptides Containing Branched-Chain Amino Acids Could Be Compensated by Free Amino Acids in the Case of Lla2125 but Not With Lla244

The proteolytic profile of Lla2125 was similar to the one of Lla244. However, the latter produced a higher number of peptides, in particular the specific peptides of cluster C5. These additional peptides were found to contain significantly lower amounts of branched-chain amino acids compared to the peptides of the other clusters. This suggests that these peptides did not effectively support the growth of prot^–^ strains, in agreement with the fact that no interaction occurred in co-cultures with Lla244, used as a donor strain (Canon et al., 2021). The two *L. lactis* prot^+^ strains also differed in the free amino acid amounts they released, especially L, I, and V: 10.0, 3.2, and 5.9 mg/L for Lla2125, respectively, against 2.7, 0, and 1.7 mg/L for Lla244 (Canon et al., 2021). Thus, the shortage of peptides containing branched-chain amino acids could have been compensated by the presence of higher amounts of free branched-chain amino acids in the case of Lla2125, but not of Lla244.

### All Proteins Are Not Suitable to Sustain Positive Interactions

We have now established that branched-chain amino acids play a central role in the positive interactions between the associated strains. Branched-chain amino acids represent 17–23% of the total amino acids in caseins and lupin proteins. Thus, theoretically, both sources of proteins could sustain the growth of the LAB strains. Unexpectedly, our study showed that lupin proteins were far less hydrolyzed by the prot^+^ strains, compared to caseins, as illustrated by the remaining intact regions shown in Figure 5. This result suggests that their potential cleavage sites are less accessible to hydrolysis, which could be explained by their globular shape, compared to the caseins, which are, in contrast, largely unfolded and thus easily hydrolyzed. Such differences in protein hydrolysis have also been observed among whey proteins, α-lactalbumin and β-lactoglobulin, the latter having a more compact globular shape and being less hydrolyzed than caseins by *Lactobacillus bulgaricus* subsp. *delbrueckii* and *Streptococcus thermophilus* (Bertrand-Harb et al., 2003). The lack of proteolysis on lupin proteins could also be due to their glycosylation at various sites of their sequences that could hamper the action of the proteolytic enzyme(s) at the proper peptide cleavage site. The capability to hydrolyze lupin proteins was both species- and strain- dependent since Efa2412 was more efficient than Lla244, which was itself more efficient than Lla2125.

To summarize, we identified three required criteria to favor positive interactions between prot^+^ and prot^–^ LAB strains: (i) the proteolytic profile of prot^+^ LAB strain, (ii) the protein composition, and (iii) the protein quaternary structure. This study gives new insight into the mechanisms that rule LAB interactions, and offers keys to improve or design LAB starters to ferment new food products.

## Data Availability Statement

The raw data supporting the conclusions of this article will be made available by the authors, without undue reservation.

## Author Contributions

FC performed the experimental study. VB-B and JJ performed the MS analyses. FC, AT, and VG wrote the manuscript. All authors contributed to the conception and design of the study, to manuscript revision, and approved the submitted version.

## Conflict of Interest

The authors declare that the research was conducted in the absence of any commercial or financial relationships that could be construed as a potential conflict of interest.

## Publisher’s Note

All claims expressed in this article are solely those of the authors and do not necessarily represent those of their affiliated organizations, or those of the publisher, the editors and the reviewers. Any product that may be evaluated in this article, or claim that may be made by its manufacturer, is not guaranteed or endorsed by the publisher.

## Footnotes

1
https://collection-cirmbia.fr/

2
https://www.uniprot.org/

3
http://bioware.ucd.ie/peptigram/

## References

[B1] Aller K. Adamberg K. Timarova V. Seiman A. Feštšenko D. Vilu R. (2014). Nutritional requirements and media development for *Lactococcus lactis* IL1403. *Appl. Microbiol. Biotechnol.* 98 5871–5881. 10.1007/s00253-014-5641-7 24626960

[B2] Bellengier P. Richard J. Foucaud C. (1997). Associative growth of *Lactococcus lactis* and *Leuconostoc mesenteroides* strains in milk. *J. Dairy Sci.* 80 1520–1527. 10.3168/jds.S0022-0302(97)76081-8

[B3] Berntsson R. P.-A. Thunnissen A.-M. W. H. Poolman B. Slotboom D.-J. (2011). Importance of a hydrophobic pocket for peptide binding in lactococcal OppA^▽^. *J. Bacteriol.* 193 4254–4256. 10.1128/JB.00447-11 21665971 PMC3147668

[B4] Bertrand-Harb C. Ivanova I. V. Dalgalarrondo M. Haertllé T. (2003). Evolution of β-lactoglobulin and α-lactalbumin content during yoghurt fermentation. *Int. Dairy J.* 13 39–45. 10.1016/S0958-6946(02)00140-1

[B5] Bingeman T. S. Perlman D. H. Storey D. G. Lewis I. A. (2017). Digestomics: an emerging strategy for comprehensive analysis of protein catabolism. *Curr. Opin. Biotechnol.* 43 134–140. 10.1016/j.copbio.2016.11.004 28025112

[B6] Canon F. Maillard M.-B. Henry G. Thierry A. Gagnaire V. (2021). Positive interactions between lactic acid bacteria promoted by nitrogen-based nutritional dependencies. *Appl. Environ. Microbiol.* 87:e0105521. 10.1128/AEM.01055-21 34347516 PMC8478457

[B7] Canon F. Nidelet T. Guédon E. Thierry A. Gagnaire V. (2020). Understanding the mechanisms of positive microbial interactions that benefit lactic acid bacteria co-cultures. *Front. Microbiol.* 11:2088. 10.3389/fmicb.2020.02088 33013761 PMC7500094

[B8] Church F. C. Swaisgood H. E. Porter D. H. Catignani G. L. (1983). Spectrophotometric assay using o-phthaldialdehyde for determination of proteolysis in milk and isolated milk proteins. *J. Dairy Sci.* 66 1219–1227. 10.3168/jds.S0022-0302(83)81926-2

[B9] Cocaign-Bousquet M. Garrigues C. Novak L. Lindley N. D. Loublere P. (1995). Rational development of a simple synthetic medium for the sustained growth of *Lactococcus lactis*. *J. Appl. Bacteriol.* 79 108–116. 10.1111/j.1365-2672.1995.tb03131.x

[B10] Deglaire A. Oliveira S. D. Jardin J. Briard-Bion V. Kroell F. Emily M. (2019). Impact of human milk pasteurization on the kinetics of peptide release during in vitro dynamic digestion at the preterm newborn stage. *Food Chem.* 281 294–303. 10.1016/j.foodchem.2018.12.086 30658760

[B11] Detmers F. J. M. Kunji E. R. S. Lanfermeijer F. C. Poolman B. Konings W. N. (1998). Kinetics and specificity of peptide uptake by the oligopeptide transport system of *Lactococcus lactis*. *Biochemistry* 37 16671–16679. 10.1021/bi981712t 9843435

[B12] Doeven M. K. Abele R. Tampé R. Poolman B. (2004). The binding specificity of OppA determines the selectivity of the oligopeptide ATP-binding cassette transporter. *J. Biol. Chem.* 279 32301–32307. 10.1074/jbc.M404343200 15169767

[B13] Doeven M. K. Kok J. Poolman B. (2005). Specificity and selectivity determinants of peptide transport in *Lactococcus lactis* and other microorganisms. *Mol. Microbiol.* 57 640–649. 10.1111/j.1365-2958.2005.04698.x 16045610

[B14] Flambard B. Helinck S. Richard J. Juillard V. (1998). The contribution of caseins to the amino acid supply for *Lactococcus lactis* depends on the type of cell envelope proteinase. *Appl. Environ. Microbiol.* 64 1991–1996. 10.1128/AEM.64.6.1991-1996.1998 9603805 PMC106269

[B15] Foucaud C. Hemme D. Desmazeaud M. (2001). Peptide utilization by *Lactococcus lactis* and *Leuconostoc mesenteroides*. *Lett. Appl. Microbiol.* 32 20–25. 10.1111/j.1472-765X.2001.00852.x 11169036

[B16] Gobbetti M. (1998). The sourdough microflora: interactions of lactic acid bacteria and yeasts. *Trends Food Sci. Technol.* 9 267–274. 10.1016/S0924-2244(98)00053-3

[B17] Guédon E. Renault P. Ehrlich S. D. Delorme C. (2001). Transcriptional pattern of genes coding for the proteolytic system of *Lactococcus lactis* and evidence for coordinated regulation of key enzymes by peptide supply. *J. Bacteriol.* 183 3614–3622. 10.1128/JB.183.12.3614-3622.2001 11371525 PMC95238

[B18] Guillot A. Boulay M. Chambellon É Gitton C. Monnet V. Juillard V. (2016). Mass spectrometry analysis of the extracellular peptidome of *Lactococcus lactis*: lines of evidence for the coexistence of extracellular protein hydrolysis and intracellular peptide excretion. *J. Proteome Res.* 15 3214–3224. 10.1021/acs.jproteome.6b00424 27439475

[B19] Ji D. Ma J. Xu M. Agyei D. (2021). Cell-envelope proteinases from lactic acid bacteria: biochemical features and biotechnological applications. *Comp. Rev. Food Sci. Food Saf.* 20 369–400. 10.1111/1541-4337.12676 33443792

[B20] Juillard V. Furlan S. Foucaud C. Richard J. (1996). Mixed cultures of proteinase-positive and proteinase-negative strains of *Lactococcus lactis* in milk. *J. Dairy Sci.* 79 964–970. 10.3168/jds.S0022-0302(96)76447-0

[B21] Juillard V. Guillot A. Le Bars D. Gripon J.-C. (1998). Specificity of milk peptide utilization by *Lactococcus lactis*. *Appl. Environ. Microbiol.* 64 1230–1236. 9546157 10.1128/aem.64.4.1230-1236.1998 PMC106134

[B22] Juillard V. Le Bars D. Kunji E. R. Konings W. N. Gripon J. C. Richard J. (1995). Oligopeptides are the main source of nitrogen for *Lactococcus lactis* during growth in milk. *Appl. Environ. Microbiol.* 61 3024–3030. 10.1128/aem.61.8.3024-3030.1995 7487034 PMC167578

[B23] Langella O. Valot B. Balliau T. Blein-Nicolas M. Bonhomme L. Zivy M. (2017). X!TandemPipeline: a tool to manage sequence redundancy for protein inference and phosphosite identification. *J. Proteome Res.* 16 494–503. 10.1021/acs.jproteome.6b00632 27990826

[B24] Ma C. Cheng G. Liu Z. Gong G. Chen Z. (2016). Determination of the essential nutrients required for milk fermentation by Lactobacillus plantarum. *LWT Food Sci. Technol.* 65 884–889. 10.1016/j.lwt.2015.09.003

[B25] Manguy J. Jehl P. Dillon E. T. Davey N. E. Shields D. C. Holton T. A. (2017). Peptigram: a web-based application for peptidomics data visualization. *J. Proteome Res.* 16 712–719. 10.1021/acs.jproteome.6b00751 27997202

[B26] Ponomarova O. Gabrielli N. Sévin D. C. Mülleder M. Zirngibl K. Bulyha K. (2017). Yeast creates a niche for symbiotic lactic acid bacteria through nitrogen overflow. *Cell Syst.* 5 345–357.e6. 10.1016/j.cels.2017.09.002 28964698 PMC5660601

[B27] Proust L. Sourabié A. Pedersen M. Besançon I. Haudebourg E. Monnet V. (2019). Insights into the complexity of yeast extract peptides and their utilization by *Streptococcus thermophilus*. *Front. Microbiol.* 10:906. 10.3389/fmicb.2019.00906 31133999 PMC6524704

[B28] R Core Team (2020). *R: A Language and Environment for Statistical Computing.* Vienna: R Foundation for Statistical Computing.

[B29] Saguir F. M. Campos I. E. L. de Nadra M. C. M. (2008). Utilization of amino acids and dipeptides by *Lactobacillus plantarum* from orange in nutritionally stressed conditions. *J. Appl. Microbiol.* 104 1597–1604. 10.1111/j.1365-2672.2007.03708.x 18217938

[B30] Savijoki K. Ingmer H. Varmanen P. (2006). Proteolytic systems of lactic acid bacteria. *Appl. Microbiol. Biotechnol.* 71 394–406. 10.1007/s00253-006-0427-1 16628446

[B31] Settachaimongkon S. Nout M. J. R. Antunes Fernandes E. C. Hettinga K. A. Vervoort J. M. van Hooijdonk T. C. M. (2014). Influence of different proteolytic strains of *Streptococcus thermophilus* in co-culture with *Lactobacillus delbrueckii* subsp. bulgaricus on the metabolite profile of set-yoghurt. *Int. J. Food Microbiol.* 177 29–36. 10.1016/j.ijfoodmicro.2014.02.008 24598513

[B32] Teusink B. Molenaar D. (2017). Systems biology of lactic acid bacteria: for food and thought. *Curr. Opin. Syst. Biol.* 6 7–13. 10.1016/j.coisb.2017.07.005 32954057 PMC7489361

[B33] Valot B. Langella O. Nano E. Zivy M. (2011). MassChroQ: a versatile tool for mass spectrometry quantification. *Proteomics* 11 3572–3577. 10.1002/pmic.201100120 21751374

[B34] van den Berg R. A. Hoefsloot H. C. Westerhuis J. A. Smilde A. K. van der Werf M. J. (2006). Centering, scaling, and transformations: improving the biological information content of metabolomics data. *BMC Genomics* 7:142. 10.1186/1471-2164-7-142 16762068 PMC1534033

[B35] Worsztynowicz P. Schmidt A. O. Białas W. Grajek W. (2019). Identification and partial characterization of proteolytic activity of *Enterococcus faecalis* relevant to their application in dairy industry. *Acta Biochim. Pol.* 66 61–69. 10.18388/abp.2018_2714 30726306

[B36] Xiao T. Yan A. Huang J. Jorgensen E. Shah N. P. (2020). Comparative peptidomic and metatranscriptomic analyses reveal improved gamma-amino butyric acid production machinery in *Levilactobacillus brevis* Strain NPS-QW 145 cocultured with Streptococcus thermophilus strain ASCC1275 during milk fermentation. *Appl. Environ. Microbiol.* 87:e01985-20. 10.1128/aem.01985-20 33067198 PMC7755244

